# Synthesis and Evaluation of Novel α-Aminoamides Containing Benzoheterocyclic Moiety for the Treatment of Pain

**DOI:** 10.3390/molecules26061716

**Published:** 2021-03-19

**Authors:** Kun Tong, Ruotian Zhang, Fengzhi Ren, Tao Zhang, Junlin He, Jingchao Cheng, Zixing Yu, Fengxia Ren, Yatong Zhang, Weiguo Shi

**Affiliations:** 1 State Key Laboratory of Toxicology and Medical Countermeasures, Beijing Institute of Pharmacology & Toxicology, 27 Tai-Ping Road, Beijing 100850, China; tongkq@126.com (K.T.); believe890521@163.com (T.Z.); hejunlin@bmi.ac.cn (J.H.); chengjingchao3017@126.com (J.C.); yuyuzx1990@163.com (Z.Y.); ren2019victory@126.com (F.R.); 2 Hebei University of Science & Technology, 26 Yuxiang Street, Shijiazhuang City 050018, China; zrtian18@163.com; 3 New Drug Research & Development Co., Ltd., North China Pharmaceutical Group Corporation, Shijiazhuang City 050015, China; rfzhhj@163.com

**Keywords:** α-aminoamides, sodium channel blocker, Na_v_1.7, Na_v_1.8, dual channel inhibitors

## Abstract

Novel α-aminoamide derivatives containing different benzoheterocyclics moiety were synthesized and evaluated as voltage-gated sodium ion channels blocks the treatment of pain. Compounds **6a**, **6e**, and **6f** containing the benzofuran group displayed more potent in vivo analgesic activity than ralfinamide in both the formalin test and the writhing assay. Interestingly, they also exhibited potent in vitro anti-Na_v_1.7 and anti-Na_v_1.8 activity in the patch-clamp electrophysiology assay. Therefore, compounds **6a**, **6e**, and **6f**, which have inhibitory potency for two pain-related Na_v_ targets, could serve as new leads for the development of analgesic medicines.

## 1. Introduction

Chronic pain syndrome, such as neuropathic pain, severely affects the quality of life of patients. However, specific analgesics for managing chronic pain are an unmet clinic need [1,2,3,4,5]. Voltage-gated sodium ion channels subtype 1.7 (Na_v_1.7) and 1.8 (Na_v_1.8) have been proven as promising targets for the discovery of new drugs to treat chronic pain and numerous small-molecule inhibitors targeting Na_v_1.7 and Na_v_1.8 that have been developed in preclinical or clinical studies in recent years [6,7,8,9,10,11,12].

In our previous study [13], we synthesized a series of novel α-aminoamide analogues containing an indole ring group based on modified ralfinamide, a Na_v_1.7-selective inhibitor for the treatment of neuropathic pain [14,15,16,17]. We found that the new compounds showed robust in vivo potency but lower Na_v_1.7 inhibitory activity in vitro compared to ralfinamide, indicating that further modifications and structure–activity relationship (SAR) investigations are necessary.

We hypothesized that the H-acceptor properties (virtually missing in indole) for H-bonding will likely be key to afford a lower Na_v_1.7 selectivity. Here, different benzoheterocyclic moiety including benzimidazole, benzofuran, and quinoline groups were introduced to replace the indolyl group, and novel compounds were synthesized and evaluated as sodium ion channel inhibitors. In addition, the in vivo potencies of the compounds as painkillers were assessed in an animal model. Interestingly, the novel compounds containing the benzofuran group exhibited potent activity in the formalin assay and state-dependent inhibition of both Na_v_1.7 and Na_v_1.8, suggesting that the novel compounds might possess dual channel activity. Their exact mechanisms of action needed to be further investigated.

## 2. Results and Discussion

### 2.1. Synthesis

**Scheme 1:**
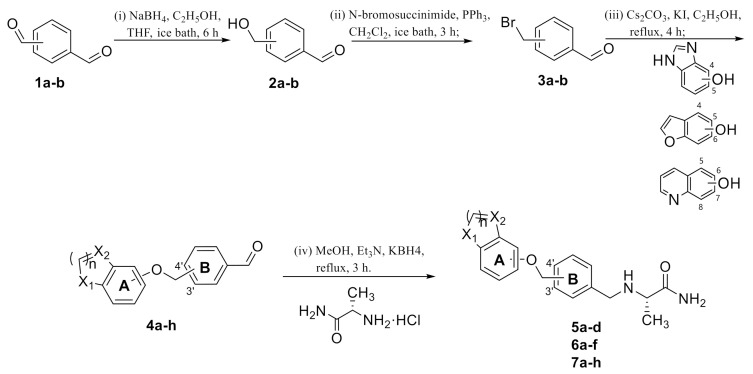
Synthesis of compounds **5a**–**d**, **6a**–**f**, and **7a**–**h**.

The synthetic route is shown in Scheme 1. The target compounds were synthesized from isophthalaldehyde (**1a**) or terephthalaldehyde (**1b**), followed by the reduction of one formyl group with sodium borohydride to obtain 3-(hydroxymethyl)benzaldehyde (**2a**) and 4-(hydroxymethyl)benzaldehyde (**2b**), respectively. Then, compounds **2a** and **2b** were converted to the intermediates 3-(bromomethyl)benzaldehyde (**3a**) and 4-(bromomethyl)benzaldehyde (**3b**) by bromination, respectively. Compounds **3a** and **3b** were etherified with a different hydroxylbenzoaromatic ring (benzimidazole, benzofuran, or quinoline) by a Williamson reaction to obtain compounds **4a–h**. Finally, compounds **4a–4h** were converted to the target α-aminoamide derivatives **5a–d**, **6a–f**, and **7a–h** with L-alaninamide hydrochloride by reductive amination.

### 2.2. Analgesic Effect of Synthesized Compounds in the Formalin Test

The analgesic potency of the new compounds was first tested with the formalin assay, as described previously [18]. As shown in Table 1, generally, the tested compounds containing the benzofuran group displayed more potent in vivo analgesic potency in phase 2 than others containing the benzimidazole and quinoline ones. The percentage of analgesia of compounds **6a**, **6e**, and **6f** was nearly 1.5–2 times higher than that of ralfinamide at a dosage of 10 mg/kg. The most potent compound **6a** showed a percentage of analgesia of 84%, compared to 42.4% for ralfinamide. The linked positions of the benzofuran ring (A ring) and the benzene ring (B ring) affected the potency of the compounds. The presence of linkages at positions 4 and 6 of the benzofuranyl group seemed to increase the analgesic activity.

**Figure 1:**
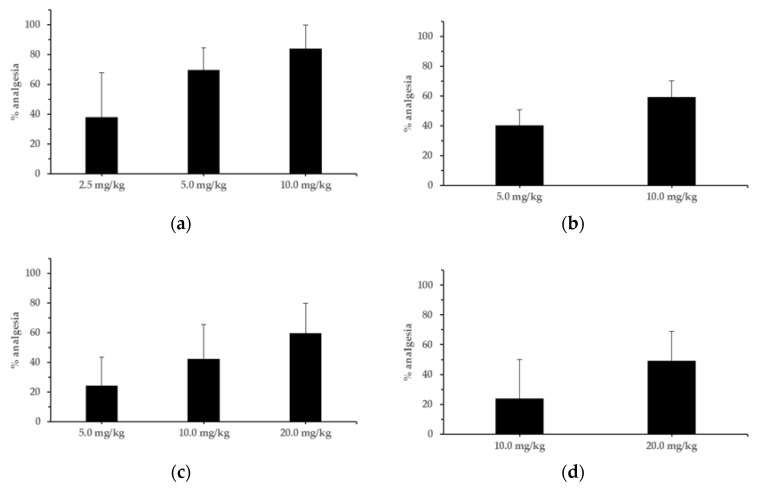
The analgesic potency of compound **6a** and ralfinamide (n = 8). (**a**). The results of the dose-effect relationship assay of compound **6a**. (**b**). The results of the oral administration assay of compound **6a**. (**c**). The results of the dose-effect relationship assay of ralfinamide. (**d**). The results of the oral administration assay of ralfinamide.

Further studies illustrated that compound **6a** was more efficient than ralfinamide in both the dose-effect relationship assays and the oral administration assays (Figure 1). Compound **6a** showed a dose-dependent activity at a dosage of 2.5 mg/kg, 5 mg/kg, and 10 mg/kg by intraperitoneal injection. The 5 mg/kg dose of compound **6a** yielded a higher (69.6%) analgesic response than 20 mg/kg ralfinamide (59.7%). The results of intragastric administration of compound **6a** by mouth also showed a higher analgesic activity than ralfinamide.

### 2.3. Analgesic Effect of Synthesized Compounds in the Acetic Acid-Induced Writhing Test

The analgesic effect of the novel compounds containing the benzofuran group was further evaluated in a mouse writhing assay, which is a method used to assess pain induced by a chemical, such as acetic acid. As shown in Table 2, compounds **6a**, **6b**, **6e**, and **6f** displayed higher activities than ralfinamide, while compounds **6c** and **6d** showed a lower potency. These compounds exhibited very similar analgesic effects in the two assays.

### 2.4. Inhibitory Activity of the Compounds and Their Effects on Tetrodotoxin (TTX)–Sensitive Inactivated Na_v_1.7 Current

The compounds were also evaluated as sodium channel inhibitors. Their effects on TTX-sensitive activated (TP−1) and inactivated (TP−2) states of Na_v_1.7 current were screened at a single dosage (10 μM), based on the IC_50_ value (7.10 ± 1.41 μM) of ralfinamide (Table 3). The compounds with the benzimidazole group showed no activity against Na_v_1.7, while compounds containing the quinoline group showed modest state-dependent inhibitory potency on Na_v_1.7, which is consistent with the result of indole compounds described in the previous study [13]. The compounds containing the benzofuran group exhibited potent inhibitory activities against Na_v_1.7. Compounds **6e** and **6f** had IC_50_ values close to that of ralfinamide, while compound **6a** was the most effective inhibitor with an IC_50_ value three times lower than that of ralfinamide (Table 4). This is consistent with the in vivo effects in the mouse formalin and writhing assays.

### 2.5. Inhibitory Activity of the Compounds against Na_v_1.8 and Na_v_1.5

For the active compounds **6a**, **6e**, and **6f,** further evaluation of their inhibitory effects on Na_v_1.8 and Na_v_1.5 was conducted. Much higher inhibition for Na_v_1.8 inactivated state Na^+^ currents was observed for these compounds, when compared to ralfinamide. Compound **6a** showed nearly nine-fold more potent against Na_v_1.8 than ralfinamide (Table 5). These findings suggest that the benzofuran moiety could play a critical role in the inhibitory activity. Meanwhile, **6a**, **6e**, and **6f** displayed moderate inhibitory activity against Na_v_1.5, similarly to ralfinamide, indicating a low selectivity over the cardiac sodium channel (Table 6).

### 2.6. Discussion

The voltage-gated sodium channel isoforms Na_v_1.7 and Na_v_1.8 are attractive drug targets for novel analgesics. However, achieving selectivity for specific subtypes with small-molecule inhibitors has been challenging [19,20]. α-Aminoamide derivatives such as ralfinamide were previously developed as potential drug candidates for the specific treatment of neuropathic pain [21,22]. Studying the relationship between target selectivity and potency for novel α-aminoamide derivatives is critically needed. Thus, we synthesized a set of novel α-aminoamide derivatives to further investigate the SARs in our previous work [13]. We confirmed that the chemical moiety of the A ring affected the selectivity to Na_v_ channels and the structure needed to be further modified. Novel α-aminoamide derivatives containing different benzo-heterocyclics moiety were synthesized in this work and evaluated as voltage-gated sodium ion channels block the treatment of pain.

Our previous studies have demonstrated that some novel compounds exhibit very high in vivo potency in the mouse formalin assay compared to ralfinamide, but lower or little in vitro inhibitory activity against Na_v_1.7. Therefore, in the present study, we first assayed the activities of the novel compounds in vivo to confirm their potency as analgesics. The results of the formalin test and writhing assay revealed the most potent compounds **6a**, **6e**, and **6f** containing the benzofuran group. Consistent with our previous study, these compounds showed no analgesic effect in a mouse hotplate assay and a tail flick assay, even at a high dose of 100 mg/kg (data not shown).

Next, we assayed the anti-Na_v_1.7 in vitro activity of the new compounds by whole-cell patch-clamp electrophysiology experiments. Compounds **6a**, **6e**, and **6f** also showed a highly potent inhibitory activity against TTX-sensitive inactivated Na_v_1.7 current, which differs from the result of the previous compounds containing an indole group, demonstrating that the benzofuran moiety is a key effective functional group for Na_v_1.7 inhibition. Moreover, compounds **6a**, **6e**, and **6f** also exhibited potent anti-Na_v_1.8 activity, which is another valid drug target for treating pain. To date, no drug candidates targeting only Na_v_1.7 or Na_v_1.8 are licensed in the market. Therefore, inhibitors with a broader selectivity for both Na_v_1.7 and Na_v_1.8 ion channels might be potential drug candidates for the treatment of pain.

Replacing the benzofuran group of compounds **6a**, **6e**, and **6f** with benzimidazole or a quinoline ring decreased the in vivo potency in the formalin assay, and the in vitro inhibitory activity against Na_v_1.7. The SAR results suggested that the benzofuran group was an essential factor for maintaining the analgesic effect in vivo as well as making it a dual target inhibitor of Na_v_1.7 and Na_v_1.8 channels *in vitro*.

The novel compounds **6a**, **6e**, and **6f** could serve as new leads for the further development of candidates for treating pain. Further work evaluating the potency of new compounds for treating neuropathic pain, and the inhibitory activity against other Na_v_ isoforms concerned with pain including Na_v_1.3, Nav1.6, and Na_v_1.9, is underway in our laboratory.

## 3. Materials and Methods

### 3.1. General Information

Commercially available reagents from Labter Pharmatech (Beijing, China), Innochem (Beijing, China), Energy (Shanghai, China), Ark Pharm (Arlington Heights, IL, USA), Fluorochem (Hadfield, UK), and Acros Organics (Geel, Belgium) were used without further purification. Nuclear magnetic resonance (NMR) spectra (^1^H-NMR, 400 MHz) were recorded on a JNM-ECA−400 spectrometer (JEOL Co. Ltd., Tokyo, Japan). Infrared spectra (IR) data were recorded using a Nicolet 6700 spectrophotometer (Thermo Fisher Scientific Co., Ltd., Waltham, MA, USA). Mass spectra were obtained on an API−150 mass spectrometer (ABI Inc., Foster City, CA, USA). Thin-layer chromatography (TLC) plates and silica gel (200–300 mesh) were purchased from Qingdao Haiyang Chemical Co. Ltd., (Shandong, China). Sample purification was conducted using Combiflash Companion Preparative Chromatography (Teledyne Isco Inc., Lincoln, NE, USA).

### 3.2. Chemistry

#### 3.2.1. Synthesis of **2a** and **2b**

Synthesis of 3-(hydroxymethyl)benzaldehyde (**2a**) and 4-(hydroxymethyl)benzaldehyde (**2b**): Isophthalaldehyde (**1a**) or terephthalaldehyde (**1b**) (4.0 equivalent 10 g, 74.55 mmol) was dissolved in a mixture of ethanol (50 mL) and tetrahydrofuran (80 mL). After cooling in an ice bath, sodium borohydride (1 equivalent 0.85 g, 18.60 mmol) was added to the solution. The reaction mixture was stirred at 0 °C for at least 6 h until the disappearance of **1a** or **1b**. Then the reaction was quenched with 3 M HCl (to pH 4–5). The solution was filtered and evaporated to dryness under a vacuum. The residue was mixed with water and extracted with EtOAc (2 × 50 mL). The combined organic layers were washed with brine and dried over anhydrous sodium sulfate. After filtration, the solvent was evaporated to obtain a crude product. The crude product was purified by flash column chromatography (petroleum ether: EtOAc = 3:1) to afford 3-(hydroxymethyl)benzaldehyde (**2a**) or 4-(hydroxymethyl)benzaldehyde (**2b**) as a colorless and transparent liquid.

#### 3.2.2. Synthesis of **3a** and **3b**

Synthesis of 3-(bromomethyl)benzaldehyde (**3a**) or 4-(bromomethyl)benzaldehyde (**3b**): Compound **2a** or **2b** (2.50 g, 18.36 mmol) was dissolved in CH_2_Cl_2_ (40 mL), and *n*-bromosuccinimide (4.90 g, 27.53 mmol) was added to the solution. After cooling in an ice bath, PPh_3_ (9.63 g, 36.72 mmol) was added to the solution. The mixture was stirred at room temperature for at least 3 h. The reaction mixture was filtered, and water (40 mL) was added to the filtrate. The solution was extracted with CH_2_Cl_2_ (2 × 20 mL). The organic layers were dried over anhydrous sodium sulfate, and the solvent was evaporated under a reduced pressure to obtain a residue. The residue was purified by silica gel column chromatography (petroleum ether: EtOAc = 10:1) to afford 3-(bromomethyl)benzaldehyde (**3a**) or 4-(bromomethyl)benzaldehyde (**3b**) as a white porous solid.

#### 3.2.3. Synthesis of **4a–h**

A mixture of 4-hydroxybenzenfuran, 5-hydroxybenzenfuran, or 6-hydroxybenzenfuran or other hydroxylbenzoaromatics (0.52 g, 3.88 mmol) and Cs_2_CO_3_ (1.31 g, 4.03 mmol) in ethanol (30 mL) was stirred at room temperature for 1 h. Then, compound 3a or 3b (0.92 g, 4.66 mmol) and kalium iodidum (0.10 g, 0.58 mmol) were added to the solution, and the mixture was refluxed for at least 4 h, until the reaction was completed. The mixture was evaporated under a reduced pressure to obtain a pale yellow solid. Then it was mixed with water and extracted with EtOAc (2 × 15 mL). The organic layers were washed with water and brine and dried over anhydrous sodium sulfate. The solvent was evaporated under a reduced pressure. The residue was purified by flash column chromatography (petroleum ether:EtOAc = 10:1) to obtain compound **4a**–**h** as a pale yellow oil.

#### 3.2.4. Synthesis of **5a–d**, **6a−6f**, **7a−7h**

A mixture of L-alaninamide hydrochloride (0.53 g, 4.28 mmol) and Et_3_N (0.87 g, 8.57 mmol) in absolute methanol (30 mL) was stirred at room temperature for 1 h. To the solution, compound **4a**–**h** (0.72 g, 2.86 mmol) was added. After stirring for another 2 h, potassium borohydride (0.92 g, 17.14 mmol) was added to the solution, and the solution was refluxed for 3 h. The solvent was evaporated under a reduced pressure to obtain a pale yellow solid. The residue was purified by silica gel column chromatography (MeOH/CH_2_Cl_2_, 0‒5%) to afford compound **5a**–**d**, **6a**–**f**, **7a**–**h** as a white solid.

##### (*S*)-2-((3-(((1H-Benzo[d]imidazol-4-yl)oxy)methyl)benzyl)amino)propanamide (**5a**)

Yield: 74%. IR (KBr, cm^−1^) *ν* 3369, 3143, 2953, 2806, 2580, 1687 (C=O), 1622 (C=N), 1552, 1514. ^1^H-NMR (400 MHz, DMSO-*d*_6_, ppm) δ_H_ 12.75 (s, 1H, NH, imidazole), 8.11 (s, 1H, H_Ar_), 7.50 (br s, 1H, CONH_2_), 7.30–7.38 (m, 4H, H_Ar_), 7.04–7.17 (m, 3H, H_Ar_, CONH_2_), 6.81 (br s, 1H, H_Ar_), 5.30 (s, 2H, OCH_2_), 3.71 (d, *J* = 13.6 Hz, 1H, Ar-CH_2_), 3.58 (d, *J* = 13.6 Hz, 1H, Ar-CH_2_), 3.04 (q, *J* = 7.0 Hz, 1H, CH-CH_3_), 1.14 (d, *J* = 7.0 Hz, 3H, CH_3_). ^13^C-NMR (100 MHz, DMSO-*d*_6,_ ppm) δ_C_ 170.3 (C=O), 146.3, 139.8, 136.7, 132.5, 132.0, 129.8, 129.2, 128.7, 127.8, 127.0, 121.5, 107.7, and 106.7 (13C, Ar-C), 69.7 (CH_2_), 54.6 (CH), 48.3 (CH_2_), 16.0 (CH_3_). HR-ESI MS *m/z* 325.1659 [M + H]^+^.

##### (*S*)-2-((4-(((1H-Benzo[d]imidazol-4-yl)oxy)methyl)benzyl)amino)propanamide (**5b**)

Yield: 88%. IR (KBr, cm^−1^) *ν* 3402, 3115, 3086, 2973, 2774, 2566, 1721 (C=O), 1621 (C=N), 1512, 1496. ^1^H-NMR (400 MHz, DMSO-*d*_6_, ppm) δ_H_ 12.74 (s, 1H, NH, imidazole), 8.10 (s, 1H, H_Ar_), 7.46 (m, 2H, H_Ar_, CONH_2_), 7.35‒7.37 (m, 3H, H_Ar_), 7.04–7.16 (m, 3H, H_Ar_, CONH_2_), 6.79 (br s, 1H, H_Ar_), 5.29 (s, 2H, OCH_2_), 3.70 (d, *J* = 13.5 Hz, 1H, Ar-CH_2_), 3.56 (d, *J* = 13.5 Hz, 1H, Ar-CH_2_), 3.02 (q, *J* = 7.0 Hz, 1H, CH-CH_3_), 1.13 (d, *J* = 7.0 Hz, 3H, CH_3_). ^13^C-NMR (100 MHz, DMSO-*d*_6,_ ppm) δ_C_ 170.4 (C=O), 146.3, 139.9, 136.9, 132.4, 131.5, 130.4, 127.7, 127.0, 121.5, 107.7, and 106.7 (13C, Ar-C), 69.6 (CH_2_), 54.6 (CH), 48.1 (CH_2_), 16.0 (CH_3_). HR-ESI MS *m/z* 325.1659 [M + H]^+^.

##### (*S*)-2-((3-(((1H-Benzo[d]imidazol-5-yl)oxy)methyl)benzyl)amino)propanamide (**5c**)

Yield: 54%. IR (KBr, cm^−1^) *ν* 3385, 3254, 3084, 2997, 2771, 2552, 1688 (C=O), 1635 (C=N), 1530, 1448. ^1^H-NMR (400 MHz, DMSO-*d*_6_, ppm) δ_H_ 12.30 (br s, 1H, NH, imidazole), 8.09 (s, 1H, H_Ar_), 7.05–7.45 (m, 8H, H_Ar_, CONH_2_), 6.89 (d, *J* = 8.4 Hz, 1H, H_Ar_), 5.11 (s, 2H, OCH_2_), 3.71 (d, *J* = 13.5 Hz, 1H, Ar-CH_2_), 3.57 (d, *J* = 13.5 Hz, 1H, Ar-CH_2_), 3.04 (q, *J* = 7.0 Hz, 1H, CH-CH_3_), 1.14 (d, *J* = 7.0 Hz, 3H, CH_3_). ^13^C-NMR (100 MHz, DMSO-*d*_6,_ ppm) δ_C_ 170.4 (C=O), 156.9, 139.6, 137.0, 132.2, 131.6, 129.9, 129.6, 128.9, 128.3, 125.1, 116.7, 115.4, and 97.7 (13C, Ar-C), 69.8 (CH_2_), 54.6 (CH), 48.4 (CH_2_), 16.0 (CH_3_). HR-ESI MS *m/z* 325.1659 [M + H]^+^.

##### (*S*)-2-((4-(((1H-Benzo[d]imidazol-5-yl)oxy)methyl)benzyl)amino)propanamide (**5d**)

Yield: 58%. IR (KBr, cm^−1^) *ν* 3326, 3258, 3145, 2929, 2763, 2659, 2557, 1706 (C=O), 1636 (C=N), 1513, 1493. ^1^H-NMR (400 MHz, DMSO-*d*_6_, ppm) δ_H_ 12.25 (irregular d, 1H, NH, imidazole), 8.06 (irregular d, 1H, H_Ar_), 7.24–7.53 (m, 7H), 6.86–7.07 (m, 2H, H_Ar_, CONH_2_), 5.10 (s, 2H, OCH_2_), 3.68 (d, *J* = 13.6 Hz, 1H, Ar-CH_2_), 3.54 (d, *J* = 13.6 Hz, 1H, Ar-CH_2_), 3.00 (q, *J* = 7.0 Hz, 1H, CH-CH_3_), 1.13 (d, *J* = 7.0 Hz, 3H, CH_3_). ^13^C-NMR (100 MHz, DMSO-*d*_6,_ ppm) δ_C_ 170.4 (C=O), 156.9, 139.7, 137.4, 131.6, 131.5, 130.4, 127.9, 125.1, 116.7, 115.4 and 97.7 (13C, Ar-C), 69.5 (CH_2_), 54.4 (CH), 48.1 (CH_2_), 16.0 (CH_3_). HR-ESI MS *m/z* 325.1658 [M + H]^+^.

##### (*S*)-2-((3-((Benzofuran-4-yloxy)methyl)benzyl)amino)propanamide (**6a**)

Yield: 91%. IR (KBr, cm^−1^) *ν* 3314, 3175, 2973, 2754, 1697 (C=O), 1631, 1604, 1545, 1493, 1436, 1368. ^1^H-NMR (400 MHz, DMSO-*d*_6_, ppm) δ_H_ 7.91 (d, *J* = 2.0 Hz, 1H, H_Ar_), 7.48 (br s, 1H, CONH_2_), 7.30–7.42 (m, 4H, H_Ar_), 7.19–7.26 (m, 2H, H_Ar_), 7.03 (br s, 1H, CONH_2_), 6.98 (dd, *J* = 2.2, 0.8 Hz, 1H, H_Ar_), 6.89 (dd, *J* = 7.3, 1.1 Hz, 1H, H_Ar_), 5.24 (s, 2H, OCH_2_), 3.72 (d, *J* = 13.5 Hz, 1H, Ar-CH_2_), 3.58 (d, *J* = 13.5 Hz, 1H, Ar-CH_2_), 3.03 (q, *J* = 6.7 Hz, 1H, CH-CH_3_), 1.14 (d, *J* = 6.7 Hz, 3H, CH_3_). ^13^C-NMR (100 MHz, DMSO-*d*_6,_ ppm) δ_C_ 170.5 (C=O), 155.7, 152.2, 144.7, 137.4, 132.2, 129.8, 129.3, 128.8, 128.1, 125.3, 117.4, 105.2, 104.7 and 104.1 (14C, Ar-C), 69.3 (CH_2_), 54.7 (CH), 48.4 (CH_2_), 16.0 (CH_3_). HR-ESI MS *m/z* 325.1547 [M + H]^+^.

##### (*S*)-2-((4-((Benzofuran-4-yloxy)methyl)benzyl)amino)propanamide (**6b**)

Yield: 56%. IR (KBr, cm^−1^) *ν* 3315, 3184, 2971, 2797, 1693 (C=O), 1606, 1544, 1494. ^1^H-NMR (400 MHz, DMSO-*d*_6_, ppm) δ_H_ 7.90 (d, *J* = 2.2 Hz, 1H, H_Ar_), 7.35–7.46 (m, 5H, H_Ar_, CONH_2_), 7.19–7.25 (m, 2H, H_Ar_), 7.04 (br s, 1H, CONH_2_), 6.96 (dd, *J* = 2.2, 0.8 Hz, 1H, H_Ar_), 6.88 (dd, *J* = 7.3, 1.3 Hz, 1H, H_Ar_), 5.23 (s, 2H, OCH_2_), 3.70 (d, *J* = 13.2 Hz, 1H, Ar-CH_2_), 3.57 (d, *J* = 13.2 Hz, 1H, Ar-CH_2_), 3.03 (m, 1H, CH-CH_3_), 1.14 (d, *J* = 7.0 Hz, 3H, CH_3_). ^13^C-NMR (100 MHz, DMSO-*d*_6,_ ppm) δ_C_ 170.4 (C=O), 155.7, 152.1, 144.8, 137.8, 131.4, 130.4, 127.7, 125.2, 117.4, 105.2, 104.7, and 104.0 (14C, Ar-C), 69.1 (CH_2_), 54.5 (CH), 48.2 (CH_2_), 16.0 (CH_3_). HR-ESI MS *m/z* 325.1546 [M + H]^+^.

##### (*S*)-2-((3-((Benzofuran-5-yloxy)methyl)benzyl)amino)propanamide (**6c**)

Yield: 87%. IR (KBr, cm^−1^) *ν* 3364, 3313, 3150, 2973, 2746, 1697 (C=O), 1613, 1570, 1462. ^1^H-NMR (400 MHz, DMSO-*d*_6_, ppm) δ_H_ 7.95 (d, *J* = 2.2 Hz, 1H, H_Ar_), 7.50 (d, *J* = 9.2 Hz, 1H, H_Ar_), 7.44 (br s, 1H, CONH_2_), 7.29–7.35 (m, 4H, H_Ar_), 7.25 (d, *J* = 2.5 Hz, 1H, H_Ar_), 7.02 (br s, 1H, CONH_2_), 6.98 (dd, *J* = 9.0, 2.5 Hz, 1H, H_Ar_), 6.88 (dd, *J* = 2.2, 0.8 Hz, 1H, H_Ar_), 5.10 (s, 2H, OCH_2_), 3.70 (d, *J* = 13.6 Hz, 1H, Ar-CH_2_), 3.56 (d, *J* = 13.6 Hz, 1H, Ar-CH_2_), 3.03 (q, *J* = 6.8 Hz, 1H, CH-CH_3_), 1.14 (d, *J* = 6.8 Hz, 3H, CH_3_). ^13^C-NMR (100 MHz, DMSO-*d*_6,_ ppm) δ_C_ 170.4 (C=O), 154.6, 149.4, 146.8, 137.7, 132.1, 129.6, 129.4, 128.7, 128.2, 127.9, 113.6, 111.8, 106.9, and 105.1 (14C, Ar-C), 69.7 (CH_2_), 54.6 (CH), 48.4 (CH_2_), 16.0 (CH_3_). HR-ESI MS *m/z* 325.1547 [M + H]^+^.

##### (*S*)-2-((4-((Benzofuran-5-yloxy)methyl)benzyl)amino)propanamide (**6d**)

Yield: 70%. IR (KBr, cm^−1^) *ν* 3307, 3265, 3167, 2967, 2798, 1690 (C=O), 1614, 1546, 1473. ^1^H-NMR (400 MHz, DMSO-*d*_6_, ppm) δ_H_ 7.94 (d, *J* = 2.2 Hz, 1H, H_Ar_), 7.49 (d, *J* = 9.0 Hz, 1H, H_Ar_), 7.33–7.42 (m, 5H, H_Ar_, CONH_2_), 7.24 (d, *J* = 2.5 Hz, 1H, H_Ar_), 7.01 (br s, 1H, CONH_2_), 6.97 (dd, *J* = 9.0, 2.5 Hz, 1H, H_Ar_), 6.88 (dd, *J* = 2.2, 0.9 Hz, 1H, H_Ar_), 5.10 (s, 2H, OCH_2_), 3.69 (d, *J* = 13.4 Hz, 1H, Ar-CH_2_), 3.55 (d, *J* = 13.4 Hz, 1H, Ar-CH_2_), 3.01 (q, *J* = 7.0 Hz, 1H, CH-CH_3_), 1.13 (d, *J* = 7.0 Hz, 3H, CH_3_). ^13^C-NMR (100 MHz, DMSO-*d*_6,_ ppm) δ_C_ 170.4 (C=O), 154.5, 149.3, 146.8, 138.1, 131.2, 130.3, 127.8, 127.7, 113.6, 111.8, 106.9, and 105.0 (14C, Ar-C), 69.4 (CH_2_), 54.5 (CH), 48.1 (CH_2_), 16.0 (CH_3_). HR-ESI MS *m/z* 325.1547 [M + H]^+^.

##### (*S*)-2-((3-((Benzofuran-6-yloxy)methyl)benzyl)amino)propanamide (**6e**)

Yield: 79%. IR (KBr, cm^−1^) *ν* 3316, 3150, 2952, 2746, 1686 (C=O), 1621, 1565, 1489. ^1^H-NMR (400 MHz, DMSO-*d*_6_, ppm) δ_H_ 7.87 (d, *J* = 2.0 Hz, 1H, H_Ar_), 7.52 (d, *J* = 9.0 Hz, 1H, H_Ar_), 7.45 (br s, 1H, CONH_2_), 7.30–7.34 (m, 5H, H_Ar_), 7.01 (br s, 1H, CONH_2_), 6.96 (dd, *J* = 8.7, 2.2 Hz, 1H, H_Ar_), 6.87 (dd, *J* = 2.2, 0.8 Hz, 1H, H_Ar_), 5.13 (s, 2H, OCH_2_), 3.70 (d, *J* = 13.6 Hz, 1H, Ar-CH_2_), 3.56 (d, *J* = 13.6 Hz, 1H, Ar-CH_2_), 3.02 (q, *J* = 7.0 Hz, 1H, CH-CH_3_), 1.13 (d, *J* = 7.0 Hz, 3H, CH_3_). ^13^C-NMR (100 MHz, DMSO-*d*_6,_ ppm) δ_C_ 170.4 (C=O), 156.5, 155.2, 145.1, 137.4, 132.1, 129.7, 129.4, 128.7, 128.3, 121.4, 120.6, 112.5, 106.5, and 97.2 (14C, Ar-C), 69.6 (CH_2_), 54.7 (CH), 48.4 (CH_2_), 16.0 (CH_3_). HR-ESI MS *m/z* 325.1546 [M + H]^+^.

##### (*S*)-2-((4-((Benzofuran-6-yloxy)methyl)benzyl)amino)propanamide (**6f**)

Yield: 82%. IR (KBr, cm^−1^) *ν* 3388, 3262, 3167, 2967, 2770, 1693 (C=O), 1621, 1544, 1489. ^1^H-NMR (400 MHz, DMSO-*d*_6_, ppm) δ_H_ 7.86 (d, *J* = 2.2 Hz, 1H, H_Ar_), 7.52 (d, *J* = 8.4 Hz, 1H, H_Ar_), 7.34–7.42 (m, 5H, H_Ar_, CONH_2_), 7.29 (d, *J* = 1.7 Hz, 1H, H_Ar_), 7.01 (br s, 1H, CONH_2_), 6.94 (dd, *J* = 8.7, 2.2 Hz, 1H, H_Ar_), 6.86 (dd, *J* = 2.2, 0.8 Hz, 1H, H_Ar_), 5.12 (s, 2H, OCH_2_), 3.69 (d, *J* = 13.6 Hz, 1H, Ar-CH_2_), 3.54 (d, *J* = 13.6 Hz, 1H, Ar-CH_2_), 3.00 (q, *J* = 6.7 Hz, 1H, CH-CH_3_), 1.13 (d, *J* = 6.7 Hz, 3H, CH_3_). ^13^C-NMR (100 MHz, DMSO-*d*_6__,_ ppm) δ_C_ 170.4 (C=O), 156.4, 155.2, 145.0, 137.8, 131.3, 130.3, 127.8, 121.4, 120.6, 112.6, 106.5, and 97.2 (14C, Ar-C), 69.2 (CH_2_), 54.5 (CH), 48.1 (CH_2_), 15.9 (CH_3_). HR-ESI MS *m/z* 325.1546 [M + H]^+^.

##### (*S*)-2-((3-((Quinolin-5-yloxy)methyl)benzyl)amino)propanamide (**7a**)

Yield: 80%. IR (KBr, cm^−1^) *ν* 3320, 3157, 2659, 2050, 1690 (C=O), 1639 (C=N), 1595, 1558. ^1^H-NMR (400 MHz, DMSO-*d*_6_, ppm) δ_H_ 8.91 (dd, *J* = 4.1, 1.6 Hz, 1H, H_Ar_), 8.58 (d, *J* = 8.4 Hz, 1H, H_Ar_), 7.68 (m, 1H, H_Ar_), 7.62 (d, *J* = 8.4 Hz, 1H, H_Ar_), 7.51–7.54 (m, 2H, H_Ar_, CONH_2_), 7.32–7.44 (m, 4H, H_Ar_), 7.18 (d, *J* = 7.6 Hz, 1H, H_Ar_), 7.04 (s, 1H, CONH_2_), 5.32 (s, 2H, OCH_2_), 3.74 (d, *J* = 13.6 Hz, 1H, Ar-CH_2_), 3.59 (d, *J* = 13.6 Hz, 1H, Ar-CH_2_), 3.04 (q, *J* = 7.0 Hz, 1H, CH-CH_3_), 1.14 (d, *J* = 7.0 Hz, 3H, CH_3_). ^13^C-NMR (100 MHz, DMSO-*d*_6__,_ ppm) δ_C_ 170.4 (C=O), 154.1, 145.5, 140.2, 139.4, 136.4, 135.1, 132.3, 130.1, 129.4, 128.8, 128.1, 121.3, 121.1, 113.3, and 109.0 (15C, Ar-C), 70.2 (CH_2_), 54.6 (CH), 48.4 (CH_2_), 16.0 (CH_3_). HR-ESI MS 336.1707 [M + H]^+^.

##### (*S*)-2-((4-((Quinolin-5-yloxy)methyl)benzyl)amino)propanamide (**7b**)

Yield: 66%. IR (KBr, cm^−1^) *ν* 3384, 3156, 2659, 2058, 1689 (C=O), 1639 (C=N), 1595, 1558. ^1^H-NMR (400 MHz, DMSO-*d*_6_, ppm) δ_H_ 8.90 (dd, *J* = 3.9, 1.4 Hz, 1H, H_Ar_), 8.56 (d, *J* = 8.4 Hz, 1H, H_Ar_), 7.67 (m, 1H, H_Ar_), 7.60 (d, *J* = 8.4 Hz, 1H, H_Ar_), 7.49–7.53 (m, 3H, H_Ar_, CONH_2_), 7.34–7.39 (m, 3H, H_Ar_), 7.17 (d, *J* = 7.6 Hz, 1H, H_Ar_), 7.02 (br s, 1H, CONH_2_), 5.31 (s, 2H, OCH_2_), 3.70 (d, *J* = 13.6 Hz, 1H, Ar-CH_2_), 3.56 (d, *J* = 13.6 Hz, 1H, Ar-CH_2_), 3.02 (q, *J* = 7.0 Hz, 1H, CH-CH_3_), 1.14 (d, *J* = 7.0 Hz, 3H, CH_3_). ^13^C-NMR (100 MHz, DMSO-*d*_6__,_ ppm) δ_C_ 170.4 (C=O), 154.0, 145.8, 139.9, 139.4, 136.8, 134.8, 131.8, 130.5, 127.9, 121.3, 121.1, 113.6, and 108.9 (15C, Ar-C), 70.0 (CH_2_), 54.6 (CH), 48.1 (CH_2_), 16.0 (CH_3_). HR-ESI MS *m/z* 336.1707 [M + H]^+^.

##### (*S*)-2-((3-((Quinolin-6-yloxy)methyl)benzyl)amino)propanamide (**7c**)

Yield: 85%. IR (KBr, cm^−1^) *ν* 3290, 3158, 2913, 2660, 2062, 1964, 1696 (C=O), 1617 (C=N), 1617, 1600. ^1^H-NMR (400 MHz, DMSO-*d*_6_, ppm) δ_H_ 8.75 (dd, *J* = 4.2, 1.7 Hz, 1H, H_Ar_), 8.25 (m, 1H, H_Ar_), 7.94 (dd, *J* = 9.5, 0.8 Hz, 1H, H_Ar_), 7.47–7.50 (m, 4H, H_Ar_, CONH_2_), 7.31–7.40 (m, 4H, H_Ar_), 7.02 (br s, 1H, CONH_2_), 5.23 (s, 2H, OCH_2_), 3.72 (d, *J* = 13.6 Hz, 1H, Ar-CH_2_), 3.58 (d, *J* = 13.6 Hz, 1H, Ar-CH_2_), 3.03 (q, *J* = 7.0 Hz, 1H, CH-CH_3_), 1.14 (d, *J* = 7.0 Hz, 3H, CH_3_). ^13^C-NMR (100 MHz, DMSO-*d*_6__,_ ppm) δ_C_ 170.4 (C=O), 157.9, 143.7, 142.4, 136.3, 134.6, 132.2, 130.3, 130.1, 130.0, 128.9, 128.5, 126.9, 123.1, 122.4, and 107.9 (15C, Ar-C), 69.9 (CH_2_), 54.6 (CH), 48.3 (CH_2_), 16.0 (CH_3_). HR-ESI MS *m/z* 336.1708 [M + H]^+^.

##### (*S*)-2-((4-((Quinolin-6-yloxy)methyl)benzyl)amino)propanamide (**7d**)

Yield: 82%. IR (KBr, cm^−1^) *ν* 3357, 3175, 3076, 2969, 2661, 1689 (C=O), 1616 (C=N), 1600, 1493. ^1^H-NMR (400 MHz, DMSO-*d*_6_, ppm) δ_H_ 8.74 (dd, *J* = 4.2, 1.7 Hz, 1H, H_Ar_), 8.24 (dd, *J* = 8.4, 1.1 Hz, 1H, H_Ar_), 7.93 (d, *J* = 8.9 Hz, 1H, H_Ar_), 7.45–7.49 (m, 5H, H_Ar_, CONH_2_), 7.36–7.38 (m, 3H, H_Ar_), 7.01 (br s, 1H, CONH_2_), 5.21 (s, 2H, OCH_2_), 3.70 (d, *J* = 13.6 Hz, 1H, Ar-CH_2_), 3.55 (d, *J* = 13.6 Hz, 1H, Ar-CH_2_), 3.01 (q, *J* = 7.0 Hz, 1H, CH-CH_3_), 1.13 (d, *J* = 7.0 Hz, 3H, CH_3_). ^13^C-NMR (100 MHz, DMSO-*d*_6__,_ ppm) δ_C_ 170.6 (C=O), 158.2, 143.8, 143.2, 137.2, 135.1, 131.7, 130.7, 130.5, 128.6, 127.2, 123.8, 122.7, 108.1 (15C, Ar-C), 69.9 (CH_2_), 54.7 (CH), 48.3 (CH_2_), 16.1 (CH_3_). HR-ESI MS *m/z* 336.1708 [M + H]^+^.

##### (*S*)-2-((3-((Quinolin-7-yloxy)methyl)benzyl)amino)propanamide (**7e**)

Yield: 81%. IR (KBr, cm^−1^) *ν* 3300, 3137, 2743, 1694 (C=O), 1643 (C=N), 1643, 1609. ^1^H-NMR (400 MHz, DMSO-*d*_6_, ppm) δ_H_ 8.81 (dd, *J* = 4.5, 2.0 Hz, 1H, H_Ar_), 8.28 (dd, *J* = 8.1, 1.7 Hz, 1H, H_Ar_), 7.90 (d, *J* = 9.0 Hz, 1H, H_Ar_), 7.48–7.50 (m, 2H, H_Ar_, CONH_2_), 7.30–7.39 (m, 6H, H_Ar_), 7.02 (br s, 1H, CONH_2_), 5.27 (s, 2H, OCH_2_), 3.71 (d, *J* = 13.6 Hz, 1H, Ar-CH_2_), 3.57 (d, *J* = 13.6 Hz, 1H, Ar-CH_2_), 3.03 (q, *J* = 7.0 Hz, 1H, CH-CH_3_), 1.14 (d, *J* = 7.0 Hz, 3H, CH_3_). ^13^C-NMR (100 MHz, DMSO-*d*_6__,_ ppm) δ_C_ 170.4 (C=O), 162.2, 145.0, 144.5, 140.7, 135.8, 132.2, 130.9, 130.3, 130.0, 129.0, 128.7, 124.3, 122.4, 119.6, and 101.3 (15C, Ar-C), 70.1 (CH_2_), 54.6 (CH), 48.3 (CH_2_), 16.0 (CH_3_). HR-ESI MS *m/z* 336.1705 [M + H]^+^.

##### (*S*)-2-((4-((Quinolin-7-yloxy)methyl)benzyl)amino)propanamide (**7f**)

Yield: 78%. IR (KBr, cm^−1^) *ν* 3365, 3177, 3020, 2719, 2661, 2396, 2085, 1689 (C=O), 1637 (C = N), 1604. ^1^H-NMR (400 MHz, DMSO-*d*_6_, ppm) δ_H_ 8.81 (dd, *J* = 4.2, 1.7 Hz, 1H, H_Ar_), 8.28 (dd, *J* = 8.2, 1.4 Hz, 1H, H_Ar_), 7.90 (d, *J* = 9.0 Hz, 1H, H_Ar_), 7.45–7.48 (m, 3H, H_Ar_, CONH_2_), 7.31–7.39 (m, 5H, H_Ar_), 7.00 (br s, 1H, CONH_2_), 5.26 (s, 2H, OCH_2_), 3.69 (d, *J* = 13.6 Hz, 1H, Ar-CH_2_), 3.55 (d, *J* = 13.6 Hz, 1H, Ar-CH_2_), 3.00 (q, *J* = 7.0 Hz, 1H, CH-CH_3_), 1.13 (d, *J* = 7.0 Hz, 3H, CH_3_). ^13^C-NMR (100 MHz, DMSO-*d*_6__,_ ppm) δ_C_ 170.8 (C=O), 163.0, 145.9, 145.1, 140.9, 137.0, 132.0, 131.5, 130.9, 128.9, 124.8, 123.0, 120.1, and 101.5 (15C, Ar-C), 70.4 (CH_2_), 54.9 (CH), 48.6 (CH_2_), 16.2 (CH_3_). HR-ESI MS *m/z* 336.1706 [M + H]^+^.

##### (*S*)-2-((3-((Quinolin-8-yloxy)methyl)benzyl)amino)propanamide (**7g**)

Yield: 96%. IR (KBr, cm^−1^) *ν* 3338, 3177, 2971, 2788, 1689 (C=O), 1631 (C=N), 1595, 1546. ^1^H-NMR (400 MHz, DMSO-*d*_6_, ppm) δ_H_ 8.86 (dd, *J* = 4.2, 1.7 Hz, 1H, H_Ar_), 8.32 (dd, *J* = 8.1, 1.7 Hz, 1H, H_Ar_), 7.49–7.57 (m, 4H, H_Ar_, CONH_2_), 7.28–7.43 (m, 5H, H_Ar_), 7.01 (br s, 1H, CONH_2_), 5.29 (s, 2H, OCH_2_), 3.72 (d, *J* = 13.6 Hz, 1H, Ar-CH_2_), 3.58 (d, *J* = 13.6 Hz, 1H, Ar-CH_2_), 3.03 (q, *J* = 7.0 Hz, 1H, CH-CH_3_), 1.13 (d, *J* = 7.0 Hz, 3H, CH_3_). ^13^C-NMR (100 MHz, DMSO-*d*_6__,_ ppm) δ_C_ 170.3 (C=O), 149.3, 145.9, 144.9, 136.2, 132.0, 130.0, 129.8, 129.6, 129.6, 128.7, 128.2, 122.8, 120.5, 115.5, and 113.8 (15C, Ar-C), 70.5 (CH_2_), 54.6 (CH), 48.4 (CH_2_), 16.0 (CH_3_). HR-ESI MS *m/z* 336.1708 [M + H]^+^.

##### (*S*)-2-((4-((Quinolin-8-yloxy)methyl)benzyl)amino)propanamide (**7h**)

Yield: 85%. IR (KBr, cm^−1^) *ν* 3323, 2958, 2746, 1689 (C=O), 1629 (C=N) 1597, 1547. ^1^H-NMR (400 MHz, DMSO-*d*_6_, ppm) δ_H_ 8.85 (dd, *J* = 4.0, 1.7 Hz, 1H, H_Ar_), 8.32 (dd, *J* = 8.4, 1.7 Hz, 1H, H_Ar_), 7.51 (dd, *J* = 8.4, 4.0 Hz, 1H, H_Ar_), 7.44–7.48 (m, 4H, H_Ar_, CONH_2_), 7.36–7.38 (m, 3H, H_Ar_), 7.26–7.31 (m, 1H, H_Ar_), 7.01 (br s, 1H, CONH_2_), 5.28 (s, 2H, OCH_2_), 3.71 (d, *J* = 13.6 Hz, 1H, Ar-CH_2_), 3.55 (d, *J* = 13.6 Hz, 1H, Ar-CH_2_), 3.02 (q, *J* = 7.0 Hz, 1H, CH-CH_3_), 1.13 (d, *J* = 7.0 Hz, 3H, CH_3_). ^13^C-NMR (100 MHz, DMSO-*d*_6__,_ ppm) δ_C_ 170.4 (C=O), 148.9, 145.6, 145.4, 136.5, 131.7, 130.4, 129.7, 129.6 128.1, 122.8, 120.4, 115.5, and 114.0 (15C, Ar-C), 70.2 (CH_2_), 54.5 (CH), 48.1 (CH_2_), 16.0 (CH_3_). HR-ESI MS *m/z* 336.1707 [M + H]^+^.

Due to the influence of the surrounding groups, the hydrogen atom of the -NH- group in these compounds was unobserved in the ^1^H-NMR spectra.

### 3.3. Formalin Test

The formalin test was carried out using the method described previously [19]. During the formalin test, Institute of Cancer Research (*ICR*; CD−1) male mice (weight: 22–25 g) were acclimated to the environment for two days before the experiment with food and water available freely. The mice were housed under a 12 h/12 h light/dark cycle. The temperature and humidity of the room were kept at 25 ± 2 °C and 50–60%, respectively. The mice were divided into a vehicle group, a ralfinamide group, and test compound groups randomly, with eight mice in each group. All compounds were dissolved in saline and administered by intraperitoneal injection (n = 8) or intragastric administration by mouth (n = 8) at 10 mg/kg. The vehicle group was given the same volume of saline.

After administration, the mice were placed into polyvinyl chloride (PVC) observation chambers. Thirty minutes later, the mice were injected subcutaneously with 20 μL of 2.7% formalin solution into the surface of their right hind paw and were returned to their PVC chambers immediately to observe their behavior. The cumulative time each mouse licked its right hind paw during phase II (15–30 min) was recorded as its painful time in phase II. The analgesic effects of the compounds were presented as the mean ± standard deviation. The data were analyzed by SPSS using one-way analysis of variance followed by Dunnett’s test. The analgesic activity of the test compounds was calculated according to the following formula.

$$\%\mathrm{Analgesia}=\frac{\mathrm{Average}\;\mathrm{Time}\;(\mathrm{Vehicle})-\mathrm{Average}\;\mathrm{Time}\;(\mathrm{Drug})}{\mathrm{Average}\;\mathrm{Time}\;(\mathrm{Vehicle})}\times \;100\%$$

### 3.4. Acetic Acid-Induced Writhing Test

The writhing test was performed according to a previously reported method [20]. The ICR (CD-1) male mice (weight: 22–25 g) were treated as for the formalin test in the same environment. The mice were divided into a vehicle group, a ralfinamide group, and test compound groups randomly, with six mice in each group. All compounds were dissolved in saline and administered by intraperitoneal injection at 10 mg/kg. The vehicle group was given the same volume of saline. After administration, the mice were placed into PVC chambers. Thirty minutes later, the mice were injected with 1% acetic acid solution at 10 mL/kg by intraperitoneal injection and placed into the observation chambers immediately to record the number of writhes each mouse had in 20 min. The results were presented as the mean ± standard deviation. The analytical method was the same as that for the formalin test, and the analgesic activity of each compound was calculated by the following formula.

$$\%\mathrm{Analgesia}=\frac{\mathrm{Average}\;\mathrm{Number}\;(\mathrm{Vehicle})-\mathrm{Average}\;\mathrm{Number}\;(\mathrm{Drug})}{\mathrm{Average}\;\mathrm{Number}\;(\mathrm{Vehicle})}\times \;100\%$$

### 3.5. Assay for hNa_v_1.7 Inhibition

The compounds were tested as hNa_v_1.7 peak current inhibitors at room temperature using human embryonic kidney 293 cells stably expressing hNa_v_1.7. The holding voltage was depolarized to 0 mV from −120 mV for 20 ms and restored to −75 mV for 8 s. After that, the membrane potential was recovered to −120 mV for 20 ms and then depolarized to 0 mV for another 20 ms. Finally, the voltage was renewed to −120 mV for 30 ms. The potential was applied every 20 s. The compounds were administered when the voltage of hNa_v_1.7 recorded by the whole-cell patch clamp was stable. Every compound concentration was tested for 5 min, and all compounds were tested at several concentrations and with multiple cells.

### 3.6. Assay for hNa_v_1.8 Inhibition

The whole-cell patch clamp assay assessing the hNa_v_1.8 current was carried out at room temperature with Chinese hamster ovary cells stably expressing hNa_v_1.8. The holding voltage of the membrane was transferred to 0 mV from −120 mV for 50 ms and then restored to −50 mV for 8 s. After that, the membrane potential was renewed to −120 mV for 20 ms, which was followed by a depolarization to 0 mV for 50 ms. Finally, the potential was recovered to the holding voltage of −120 mV for 30 ms. This process was performed every 20 s. The compounds were administered when the voltage of hNa_v_1.8 was stable. Every compound concentration was tested for 5 min, and all compounds were tested at several concentrations and with multiple cells.

### 3.7. Assay for hNa_v_1.5 Inhibition

The inhibition of the compounds to hNa_v_1.5 peak current was performed at room temperature using HEK293 cells that could express hNa_v_1.5 stably. The holding voltage of the membrane was transferred to 0 mV from −120 mV for 50 ms and then restored to −50 mV for 8 s. After that, the membrane potential was renewed to −120 mV for 20 ms, followed by a depolarization to 0 mV for 50 ms. At last, the potential was recovered to the holding voltage −120 mV for 30 ms. This progress was performed every 20 s. The compounds were administrated when the voltage of hNa_v_1.5 was stable. Every concentration was tested for 5 min and all compounds were tested at several concentrations and with multiple cells.

## 4. Conclusions

In conclusion, novel α-aminoamide derivatives containing benzo-aromatic heterocyclic groups were synthesized and evaluated as sodium channel isoform blocks for treating pain. Compounds **6a**, **6e**, and **6f** displayed a greater in vivo analgesic potency than ralfinamide in both the formalin test and the writhing assay. Interestingly, they exhibited potent both anti-Na_v_1.7 and anti-Na_v_1.8 activity in the patch-clamp electrophysiology assay. The new compounds could serve as new leads for the development of analgesic drugs.

## Figures and Tables

**Table 1:**
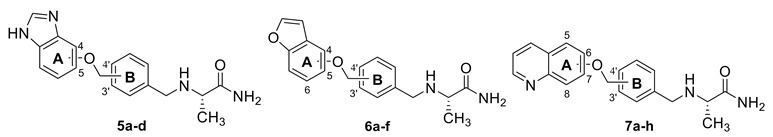
The analgesic activity of the synthesized compounds in the formalin test.

Compound	Substituted Position of the Ether Bond in the A Ring	Substituted Position of the Pharmacophore in the B Ring	% Analgesia ^a^ (10 mg/kg, *i.p.*)
**5a**	4	3′	51.0 ± 21.6 ^☆^
**5b**	4	4′	27.4 ± 19.2
**5c**	5	3′	48.8 ± 15.5 ^☆^
**5d**	5	4′	60.8 ± 32.2 ^☆^
**6a**	4	3′	84.0 ± 16.2 ^☆ *^
**6b**	4	4′	48.4 ± 26.4 ^☆^
**6c**	5	3′	30.2 ± 19.8
**6d**	5	4′	42.2 ± 16.3
**6e**	6	3′	71.7 ± 22.0 ^☆ @^
**6f**	6	4′	67.8 ± 22.7 ^☆ @^
**7a**	5	3′	48.0 ± 27.8 ^☆^
**7b**	5	4′	37.2 ± 25.3
**7c**	6	3′	81.5 ± 14.8 ^☆ *^
**7d**	6	4′	20.9 ± 18.7 ^☆^
**7e**	7	3′	30.6 ± 14.7
**7f**	7	4′	15.9 ± 17.6
**7g**	8	3′	35.6 ± 26.1
**7h**	8	4′	57.2 ± 30.1 ^☆^
Ralfinamide	—	—	42.4 ± 23.3 ^#^

^a^ Data are presented as the mean ± standard deviation. Two-tailed Student’s t test. ^☆^ *p* < 0.001 compared to vehicle. ^#^ *p* < 0.05 compared to vehicle. ^*^ *p* < 0.001 compared to ralfinamide. ^@^ *p* < 0.05 compared to ralfinamide.

**Table 2:**
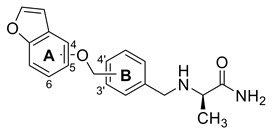
The analgesic activity of the synthesized compounds in the acetic acid-induced writhing test.

Compound	Substituted Position of the Ether Bond in the A Ring	Substituted Position of the Pharmacophore in the B Ring	% Analgesia ^a^ (10 mg/kg, *i.p.*)
**6a**	4	3′	49.7 ± 11.3 ^☆^
**6b**	4	4′	48.0 ± 5.8 ^☆^
**6c**	5	3′	11.2 ± 7.6
**6d**	5	4′	28.9 ± 8.5 ^#^
**6e**	6	3′	45.7 ± 17.2 ^☆^
**6f**	6	4′	63.2 ± 13.3 ^☆ @^
Ralfinamide	—	—	44.4 ± 15.5 ^☆^

^a^ Data are presented as the mean ± standard deviation. Two-tailed Student’s t test. ^☆^ *p* < 0.001 compared to vehicle. ^#^ *p* < 0.05 compared to vehicle. ^@^ *p* < 0.05 compared to ralfinamide.

**Table 3:** Inhibitory activity of the compounds against Na_v_1.7.

Compound	Substituted Position of the Ether Bond in the A Ring	Substituted Position of the Pharmacophore in the B Ring	Inhibitory Effect against Na_v_1.7 (%, 10 µM)	
			Activated State (TP−1)	Inactivated State (TP−2)
**5a**	4	3′	5.1 ± 1.2	10.7 ± 0.4
**5b**	4	4′	3.7 ± 2.0	1.4 ± 0.6
**5c**	5	3′	1.7 ± 0.3	2.6 ± 0.8
**5d**	5	4′	0.7 ± 0.8	1.5 ± 0.9
**6a**	4	3′	15.6 ± 0.8	61.8 ± 0.2 ^☆^
**6b**	4	4′	11.8 ± 0.3	55.5 ± 2.5 ^☆^
**6c**	5	3′	12.2 ± 0.7	48.1 ± 4.2 ^☆^
**6d**	5	4′	10.9 ± 1.1	31.8 ± 0.3 ^#^
**6e**	6	3′	11.4 ± 1.6	58.5 ± 1.8 ^☆^
**6f**	6	4′	12.4 ± 2.9	50.4 ± 3.6 ^#^
**7a**	5	3′	6.4 ± 4.9	23.8 ± 3.6
**7b**	5	4′	14.7 ± 5.4	29.8 ± 0.6 ^#^
**7c**	6	3′	8.6 ± 1.2	30.0 ± 2.9 ^#^
**7d**	6	4′	3.7 ± 0.5	13.5 ± 0.4
**7e**	7	3′	3.6 ± 3.3	47.7 ± 2.7 ^☆^
**7f**	7	4′	−1.8 ± 3.2	31.6 ± 3.4 ^#^
**7g**	8	3′	14.7 ± 0.2	46.0 ± 0.1 ^☆^
**7h**	8	4′	9.1 ± 4.3	38.6 ± 4.7 ^#^
Ralfinamide	—	—	8.1 ± 3.8	57.7 ± 3.4 ^☆^

^a^ Data are presented as the mean ± standard deviation. Two-tailed Student’s t test, ^☆^ *p* < 0.001 compared to vehicle. ^#^ *p* < 0.05 compared to vehicle.

**Table 4:** In vitro inhibitory activity of novel compounds.

Compound	Substituted Position of the Ether Bond in the A Ring	Substituted Position of the Pharmacophore in the B Ring	IC_50_ to Na_v_1.7 (µM)
**6a**	4	3′	2.3 ± 0.2
**6e**	6	3′	9.4 ± 2.7
**6f**	6	4′	8.1 ± 1.1
Ralfinamide	—	—	6.6 ± 1.6

**Table 5:** Inhibitory activity of the compounds against Na_v_1.8.

Compound	Substituted Position of the Ether bond in the A ring	Substituted Position of the Pharmacophore in the B ring	IC_50_ to Na_v_1.8 (µM)
**6a**	4	3′	4.3 ± 0.5
**6e**	6	3′	10.1 ± 1.9
**6f**	6	4′	4.8 ± 0.7
Ralfinamide	—	—	36.3 ± 8.9

**Table 6:** Inhibitory activity of the compounds against Na_v_1.5.

Compound	Substituted Position of the Ether Bond in the A Ring	Substituted Position of the Pharmacophore in the B Ring	Inhibitory Effect to Na_v_1.5 (%, 10 µM)
**6a**	4	3′	66.8 ± 1.5
**6e**	6	3′	70.1 ± 2.2
**6f**	6	4′	48.0 ± 0.1
Ralfinamide	—	—	64.6 ± 3.2

## Data Availability

The data presented in this study are available in this article.

## Sample Availability

Samples of the compounds are available from the authors.
